# Machine translation training data for English–Tshivenḓa

**DOI:** 10.1016/j.dib.2024.110898

**Published:** 2024-09-07

**Authors:** Tanja Gaustad, Cindy A. McKellar, Martin J. Puttkammer

**Affiliations:** Centre for Text Technology (CTexT), North-West University, 11 Hoffman Street, Potchefstroom, South Africa

**Keywords:** Machine translation, Natural language processing, Human language technology, Parallel corpora, Bilingual data, English, Tshivenḓa

## Abstract

This data article describes a machine translation training data set for translation between English and Tshivenḓa. The data set contains parallel, aligned English–Tshivenḓa data as well as monolingual Tshivenḓa data. The data was collected from both web crawling of multilingual South African government sites and matched documents from translators or publishing sources. Additional unique data was translated from English into Tshivenḓa by professional translators to increase the total corpus size. This article contains information about the collection and translation of the data as well as how alignments and corpus cleanup were done. The wordcounts of the corpus are also given. In addition to training machine translation systems this data can also be used for the development of other Tshivenḓa core technologies as well as for linguistic studies.

Specifications Table

**Table:** 

Subject	Computer Science
Specific subject area	Machine Translation - subfield of natural language processing/human language technology
Type of data	Aligned and monolingual text (UTF8)
Data collection	Dataset was created by combining data from three different sources: translation of English sentences into Tshivenḓa by professional translators, crawling websites with English and Tshivenḓa parallel data (government domain) and sourcing of existing data from various translators and multilingual publications. Bilingual files were aligned on sentence level and Tshivenḓa data was used for the monolingual corpus.
Data source location	Institution: Centre for Text Technology, North-West University City/Town/Region: Potchefstroom Country: South Africa
Data accessibility	Repository name: SADiLaRData identification number: - Monolingual: https://hdl.handle.net/20.500.12185/681 - Bilingual: https://hdl.handle.net/20.500.12185/682 Direct URL to data: - Monolingual: https://repo.sadilar.org/handle/20.500.12185/681 - Bilingual: https://repo.sadilar.org/handle/20.500.12185/682

## 1 Value of the Data

• This parallel and monolingual dataset can be used for the training of machine translation systems between two South African languages: English and Tshivenḓa.
• Any researcher working in the field of machine translation between these two South African languages can benefit from this data.
• This data can be used for future research and development in machine translation, as well as any other application that may benefit from bilingual, aligned data.
• Any researcher in need of monolingual Tshivenḓa data for other natural language processing research can also use the Tshivenḓa part of the parallel dataset along with the monolingual Tshivenḓa data for their research.

## 2 Background

This dataset was created as part of the Autshumato project.^1^ The Autshumato project was initiated and funded in 2007 by the South African Department of Sports, Arts and Culture (DSAC) for the purpose of developing, releasing, and supporting open-source translation technologies for the South African languages. The latest (6th) version of the project was funded by the South African Centre for Digital Language Resources (SADiLaR) and new data resources and translation resources are continuously added as they become available. This dataset was created to be used for the training of machine translation systems for the Autshumato project and to serve as a reusable linguistic resource for the development of other natural language processing applications for the resource-scarce language Tshivenḓa.

## 3 Data Description

The English–Tshivenḓa machine translation training dataset consists of two different types of data. There is a monolingual Tshivenḓa corpus containing all the Tshivenḓa data collected during the project. The second corpus contains bilingual, aligned English–Tshivenḓa segment pairs. There may be some overlap between the Tshivenḓa portion of the bilingual data and the monolingual corpus. The monolingual corpus is in a single .txt file with one sentence/text segment per line. Segments are unique, randomized to protect original document content and in UTF8 encoding. The bilingual data is in an aligned pair of .txt files with one sentence/text segment per line. Aligned sentences are on corresponding lines of the files. Table 1 contains an overview of the segments and word counts in the dataset. For the purposes of the data counts, any line that contains at least 1 word or number is counted and any word that contains at least one alpha-numeric character is counted. Loose standing punctuation was not added to the word counts.

**Table 1:** Wordcounts of bilingual and monolingual English–Tshivenḓa machine translation training data.

	Segments	English words	Tshivenḓa words
Tshivenḓa Monolingual Corpus	141,426	–	2,870,916
English–Tshivenḓa Bilingual Corpus	110,367	2,000,657	2,527,789

## 4 Experimental Design, Materials and Methods

This dataset contains data from three different sources: translation (50 %), crawling (42 %) and sourcing of existing parallel data (8 %). Translated data was created by taking documents from the South African government domain, removing any sentences that overlap with existing data and having the remainder translated by professional translators. Websites with English and Tshivenḓa data (mostly also government domain) were crawled for existing bilingual data. Already translated data was also sourced from various translators and multilingual publications.

The translated corpus was based on English government domain documents that do not have Tshivenḓa translations. These documents were divided into sentences and then all sentences were filtered to remove the sentences that overlapped with existing parallel data and each other. Sentences with many names or spelling mistakes were also removed by running them through a spelling checker and keeping only the sentences that where at least 80 % recognised by the spelling checker. Sentences were then reviewed by English speaking assistants to remove any that did not make sense without the context of the document as well as to fix grammatical errors. The corrected sentences were then sent to a professional translation company to be translated by professional English–Tshivenḓa translators. All translations were checked by assistants as an initial quality check to see if the sentence matched the original and 5 % of the data was also sent to a Tshivenḓa language expert for external quality control. This data did not need to be aligned as it was already in translated sentence pairs. Also, due to the high standards of quality control there was no need for it to be run through the data cleaning process the other data went through.

Existing bilingual data was sourced from translators and multilingual publications. This data was already directly translated and of good quality so very little preparation was needed for the documents. All the data was converted into text files, separated into sentences, tokenized and aligned with the HunAlign [1] algorithm. The aligned sentences were combined with the crawled data and run through the same final cleanup process (described below).

To create the crawled portion of the English–Tshivenḓa corpus, South African government websites where crawled using HTTrack.^2^ All documents were converted to UTF8 encoded text and analysed with the CTexT tools^3^ language identifier [2,3] to identify the language of each document since the websites contained information in all the official languages of South Africa. English and Tshivenḓa documents where then aligned on document level using a combination of document names, website structure and internal document structures. The documents cover a wide range of topics of interest to the general public. This includes education, health, communications, technology, policing, environment, human settlements, politics, law and many other fields. As the government websites cover such a large variety of topics the data is reasonably diverse although it does not contain creative writing or news topics. All the data also originates with the South African government which uses only professional translators who work with the official Tshivenḓa orthography and spelling rules.

Tshivenḓa uses 10 different diacritic symbols (*viz*. Ḓ, ḓ, Ḽ, ḽ, Ṅ, ṅ, Ṋ, ṋ, Ṱ and ṱ) as part of their writing system. Some of the documents from the web crawl did not contain any of these diacritic symbols indicating incorrect language use by the content creators or due to software used in the creation of the documents that is unable to accommodate the diacritics. Other documents had corrupted or missing diacritics due to problems with the document conversion. Examples of the badly formatted diacritics can be seen in the text box below. In both cases it was not possible to automatically insert the diacritics in the documents without creating errors in the data. For this reason, any Tshivenḓa document that did not contain correct diacritic symbols were discarded along with its aligned English document (Text Box 1).

**Table:** **Text Box 1:** Examples of badly created Tshivenḓa diacritics.

Mudzulatshidulo wa SALGA, Vhora?orobo na vharangaphan?a kha sisteme
Khantsela ya Dziminisit°a dza Pfunzo, vha t°ahisa nd°ivhadzo u ya
u †u†uwedza u ∂ihudza kha shango ¬ashu na dzhango ¬ashu.
tshimbilelane nga nila dzi shumaho dza sisieme dza
kha l iṅwe na l iṅwe l a mavundu a t ahe,

The aligned documents from the web crawl were then aligned on sentence level following the same process as was used for the sourced data. All aligned documents were automatically checked to analyse the amount of data lost during alignment. Documents that aligned badly were manually checked for errors and corrected either automatically or manually to improve alignment quality and increase the amount of data in the corpus. Documents that where incorrectly matched up during the document alignment and did not align on sentence level were discarded.

After each of the types of data had been individually processed, the aligned bilingual files were combined and put through a final clean-up process to ensure the corpus is of the best possible quality. The combined corpus was processed with the CTexT language identifier on sentence level to remove any individual sentences where the languages are not correct due to mixed language files. All duplicate aligned sentence pairs were removed since the crawled data tend to contain a great deal of duplications due to document duplication, repeated information and website menus. All line pairs containing no usable text (i.e. only numbers) were removed as is any remaining text with damaged diacritics on the Tshivenḓa side. Finally, all the sentence pairs were randomized to protect the content of the original documents. Table 2 shows the amount of data at various stages of processing for the crawled and sourced data.

**Table 2:** Overview of number of words retained and percentage of remaining data for the combined crawled and sourced data after each processing step.

Processing of combined data	Tshivenḓa Word counts	% of remaining data
Original data	29,267,752	100,00
After diacritics check	27,926,501	95,42
After alignment check	21,267,137	72,66
Unique data	1,433,897	4,90
Language Identified	1,374,438	4,70
Final cleaned data	1,251,744	4,28

All monolingual Tshivenḓa data was put through a similar process as the bilingual text except for document and sentence alignment. The final monolingual corpus contains tokenised, unique sentences which have been identified as being in the Tshivenḓa language. All lines containing broken diacritics and little usable text were removed and the lines were randomized to create the final version of the corpus.

## Limitations

This dataset is somewhat limited in both domain and size. Tshivenḓa is a resource-scarce language and one of the South African languages with the fewest speakers. Several data types commonly available for other languages, such as news, are not present or not easily findable. Therefore, although this dataset is much smaller than those of widely spoken languages, it is still a significant amount of data for the language. Most of the data was collected from government websites or translated from English versions of government documents. This means that while there is a variety of different topics in the data there are also missing elements, such as news articles and novels.

There are also ethical considerations involved when using web-crawled data, especially relating to privacy and consent. All the crawled data contained in the described dataset originates from official South African government websites that are already in the public domain and do not contain sensitive data.

## Ethics Statement

The authors have read and understood the ethical guidelines and followed the ethical requirements for publication in Data in Brief. The data set described in this article did not involve human subjects, animal experiments or any data collected from social media platforms.

## CRediT Author Statement

**Tanja Gaustad**: Supervision, Validation, Data Curation, Writing – Review & Editing, Project administration. **Cindy A McKellar**: Methodology, Data Curation, Validation, Writing – Original Draft, Writing – Review & Editing. **Martin J. Puttkammer**: Conceptualization, Funding acquisition, Data Curation, Writing – Review & Editing.

## Data Availability

Autshumato English-Tshivenḓa Parallel Corpora (Original data) (SADiLaR - Language Resource Management Agency). Autshumato Monolingual Tshivenḓa Corpus (Original data) (SADiLaR - Language Resource Management Agency).

## Footnotes

1 https://autshumato.sourceforge.io/

2 www.httrack.com

3 The Language Identifier used was updated after the cited publications, but there has not yet been an updated article published.
